# Efficacy, Safety, and Tolerability of Tivozanib in Heavily Pretreated Patients With Advanced Clear Cell Renal Cell Carcinoma

**DOI:** 10.1093/oncolo/oyae037

**Published:** 2024-03-13

**Authors:** Andrew C Johns, Matthew T Campbell, Mamie Gao, Andrew W Hahn, Zita Lim, Emily Wang, Jianjun Gao, Amishi Y Shah, Pavlos Msaouel, Nizar M Tannir

**Affiliations:** Division of Cancer Medicine, University of Texas MD Anderson Cancer Center, Houston, Texas; Department of Genitourinary Medical Oncology, University of Texas MD Anderson Cancer Center, Houston, Texas; Department of Abdominal Imaging, University of Texas MD Anderson Cancer Center, Houston, Texas; Department of Genitourinary Medical Oncology, University of Texas MD Anderson Cancer Center, Houston, Texas; Department of Genitourinary Medical Oncology, University of Texas MD Anderson Cancer Center, Houston, Texas; Pharmacy Clinical Programs, University of Texas MD Anderson Cancer Center, Houston, Texas; Department of Genitourinary Medical Oncology, University of Texas MD Anderson Cancer Center, Houston, Texas; Department of Genitourinary Medical Oncology, University of Texas MD Anderson Cancer Center, Houston, Texas; Department of Genitourinary Medical Oncology, University of Texas MD Anderson Cancer Center, Houston, Texas; Department of Genitourinary Medical Oncology, University of Texas MD Anderson Cancer Center, Houston, Texas

**Keywords:** renal cell carcinoma, tivozanib, tyrosine kinase inhibitor, VEGF blockade, sequencing

## Abstract

**Background:**

Tivozanib has been approved as a third-line or later therapy for advanced renal cell carcinoma based on the TIVO-3 trial, which was conducted before immune checkpoint therapies (ICT), cabozantinib, and lenvatinib/everolimus became incorporated in the current sequential treatment paradigm for advanced clear cell RCC (ccRCC).

**Methods:**

We performed a retrospective study of patients with advanced ccRCC treated with tivozanib at MD Anderson Cancer Center during 6/2021-7/2023. A blinded radiologist assessed tumor response by RECIST v1.1. We assessed overall response rate (ORR), clinical benefit rate (CBR) [percentage of all treated patients who achieved radiologic response or stable disease (SD) for ≥ 6 months], progression-free survival (PFS), overall survival (OS), and safety.

**Results:**

Of 30 analyzed patients, 23% had performance status ≥ 2; 47% had International Metastatic RCC Database Consortium (IMDC) poor-risk disease. Median number of prior therapies was 4 (range 1-8). All patients received prior ICT, 87% cabozantinib and 60% lenvatinib ± everolimus. Of 26 evaluable patients, 2 patients had confirmed partial response (ORR 7.7%); 5 patients had SD for ≥ 6 months (CBR 23.3%). Median PFS was 3.8 months (range 0.7-13.9); median OS was 14.1 months (range 0.3-28.5). Fifteen patients (50%) had ≥ 1 treatment-related adverse event (TRAE). There were 6 grade ≥ 3 TRAEs [hypertension, congestive heart failure (3), mucositis, and GI perforation (grade 5)].

**Conclusions:**

In this cohort of heavily pretreated patients with advanced ccRCC, tivozanib yielded a modest clinical benefit in a minority of patients who received prior ICT, cabozantinib, and lenvatinib ± everolimus. TRAEs were consistent with previously published reports.

## Teaser

This article describes the outcomes of patients who were treated with tivozanib for advanced renal cell carcinoma (RCC) and provides data to guide sequencing of therapies and patient selection for treatment with tivozanib in the current RCC treatment paradigm.

**Implications for Practice**

Tivozanib has modest efficacy in heavily pretreated patients with advanced renal cell carcinoma. Patients with relatively indolent disease and prior prolonged treatment with one or more lines of tyrosine kinase inhibitor therapy are most likely to benefit from tivozanib.

## Introduction

Tivozanib is a tyrosine kinase inhibitor (TKI) that predominantly inhibits the vascular endothelial growth factor receptors (VEGFR) with minimal inhibition of other tyrosine kinases. Tivozanib achieves blockade of the VEGFR-1, 2, and 3 receptors at very low concentrations, allowing for relatively low doses that result in a limited off-target toxicity profile compared with other drugs in its class.^1^ Tivozanib was approved by the Food and Drug Administration (FDA) in 2021 for the treatment of advanced RCC in third-line or later based on the phase 3, international, multicenter TIVO-3 trial.^2,3^ In that study, patients previously treated with at least 2 systemic therapies (including at least one VEGFR TKI) were randomized 1:1 to receive either tivozanib or sorafenib between May 2016 and August 2017. Progression-free survival (PFS), which was the primary endpoint of the study, was 5.6 months with tivozanib vs. 3.9 months with sorafenib (hazard ratio 0.73, 95% CI 0·56-0·94; *P* = .016). The overall response rate (ORR) by RECIST v1.1 for tivozanib was 18%, with stable disease reported for 55% of patients. Median duration of exposure for tivozanib was 197 days. The most common grades 3-4 adverse event reported with tivozanib was hypertension (20%), and rates of other TKI-associated adverse events including hand-foot skin reaction and diarrhea substantially decreased among patients receiving tivozanib.

The treatment landscape for RCC has rapidly evolved over the past few years with several approved therapies entering the first-line space since the TIVO-3 trial completed accrual.^4-7^ It is unclear whether the benefit of tivozanib extends to patients who have previously been treated with contemporary therapies such as dual-checkpoint inhibitors (nivolumab and ipilimumab), TKI-checkpoint inhibitor combinations, or later-generation TKIs, particularly cabozantinib and lenvatinib. In the TIVO-3 trial, only 26% of patients had received prior immune checkpoint therapy (ICT), and no patients had received more than 3 lines of prior therapy, which limits the applicability of the trial’s findings to current practice. Furthermore, based on the timing of the US FDA approvals for cabozantinib (4/2016)^8^ and lenvatinib (5/2016),^9^ it is unlikely that a significant number of patients treated on the TIVO-3 trial had previously received either of these agents, and TIVO-3 did not report details on prior therapies. In the present study, we describe the outcomes of patients who were treated with tivozanib for advanced RCC at our center and provide data which may guide sequencing of therapies and patient selection for treatment with tivozanib in the current RCC treatment paradigm.

## Methods

This is a single-center, retrospective study of patients treated with tivozanib for advanced clear cell RCC at the University of Texas MD Anderson Cancer Center (MDACC) between March 2021 and July 2023. The study was approved by the MDACC Institutional Review Board (protocol PA16-0736). The electronic medical records and pharmacy records were queried for a comprehensive list of all patients who were prescribed tivozanib during this time duration. Patients who were not confirmed to have started treatment or did not have adequate follow-up information were excluded from the analysis. Baseline patient demographic and clinical characteristics, including International Metastatic RCC Database Consortium (IMDC) risk score at the time of initiation of tivozanib therapy, prior RCC therapies, sites of metastatic disease, and dates of treatment with tivozanib, were abstracted from the electronic medical records. A blinded, board-certified radiologist (M.G.) reviewed and confirmed all cases of major response [complete response (CR) or partial response (PR)] or stable disease (SD) using RECIST v1.1. ORR, clinical benefit rate (CBR; defined as percentage of treated patients with CR, PR, or SD for at least 6 months), and time on treatment (TOT) were analyzed using descriptive statistics. PFS and overall survival (OS) were characterized using the Kaplan-Meier method (GraphPad Prism, v10, Boston, MA, USA). Median follow-up time was estimated using the reverse Kaplan-Meier method. For the PFS analysis, patients were censored if they stopped treatment for non-progression or if they remained on treatment at the time of last follow-up. For the OS analysis, patients were censored at the time of last follow-up if they remained alive at the time of analysis or if death status could not be ascertained. Adverse events related to tivozanib were abstracted and graded by a physician (A.C.J.) using CTCAE v5.0 criteria based on available documentation in the medical records.

## Results

### Baseline Characteristics

Thirty patients were included in our analysis. Baseline characteristics are shown in Table 1. Patients were heavily pretreated with median of 4 prior lines of therapy, and only 2 (6.7%) patients had not received either cabozantinib or lenvatinib. All patients had previously received ICT. More than half of patients had at least one site of bone metastasis, and 46.7% of patients had poor-risk disease by IMDC criteria.

**Table 1.** Baseline patient characteristics.

Characteristic	Number of patients (%) *N* = 30
Age (median, range)	66 (43-80)
Gender	
Male	23 (76.7)
Female	7 (23.3)
ECOG performance status	
0-1	20 (66.7)
2	6 (20)
3	1 (3.3)
Unknown	3 (10)
IMDC risk group	
Intermediate risk	16 (53.3)
Poor risk	14 (46.7)
Prior nephrectomy	24 (80)
Sarcomatoid/rhabdoid histologic features	4 (13.3)
Number of prior therapies (median, range)	4 (1-8)
≥1	30 (100)
≥2	29 (96.7)
≥3	25 (83.3)
≥4	16 (53.3)
≥5	8 (26.7)
Any prior checkpoint inhibitor	30 (100)
Nivolumab + ipilimumab	9 (30)
Any prior tyrosine kinase inhibitor	30 (100)
Pazopanib	10 (33.3)
Axitinib	12 (40)
Cabozantinib	26 (86.7)
Lenvatinib	18 (60)
Everolimus	18 (60)
Other	14 (46.7)
≥3 Metastatic sites	25 (83.3)
Lung	26 (86.7)
Lymph nodes	23 (76.7)
Bone	17 (56.7)
Liver	8 (26.7)
Brain	7 (23.3)
Other	21 (70)
Tivozanib starting dose	
1.34 mg/day (3 weeks on, 1 week off)	23 (76.7)
0.89 mg/day (3 weeks on, 1 week off)	7 (23.3)

### Efficacy

**Figure 1.**
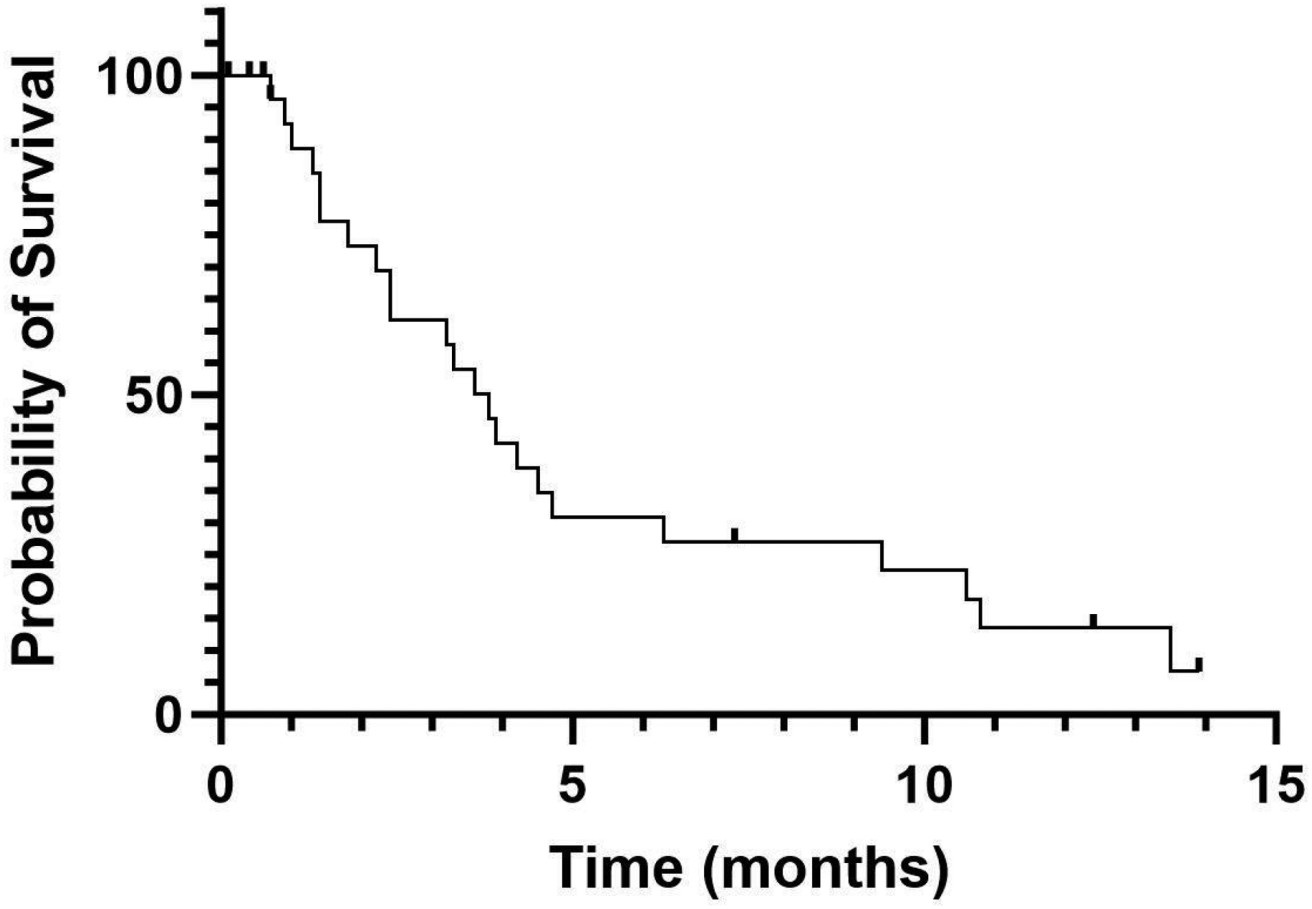
Progression-free survival. Kaplan-Meier curve showing progression-free survival (PFS) for patients treated with tivozanib. Median PFS was 3.8 months. Patients were censored if they stopped treatment for non-progression or remained on treatment at the time of analysis.

**Figure 2.**
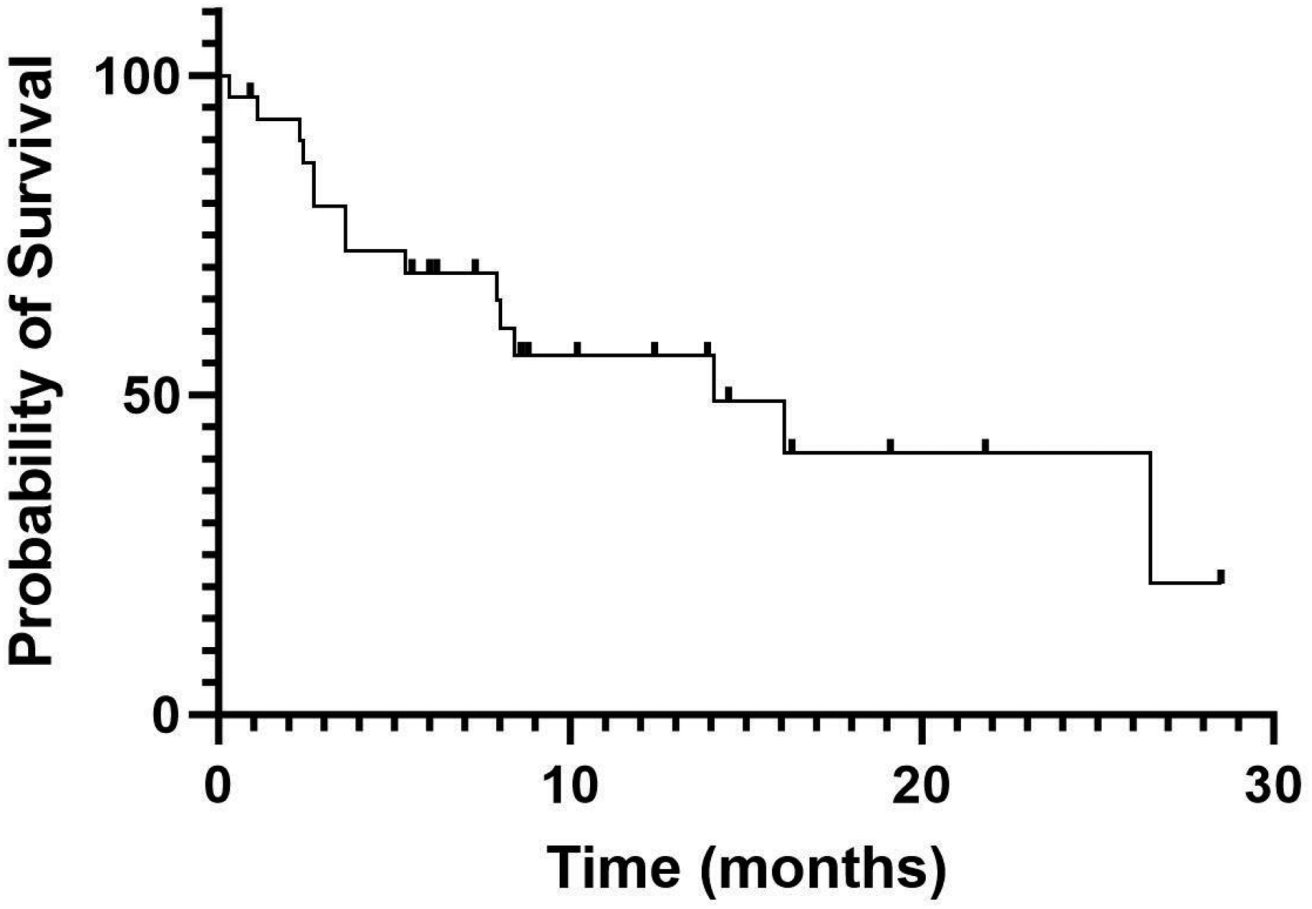
Overall survival. Kaplan-Meier curve showing overall survival (OS) for patients treated with tivozanib. Median OS was 14.1 months. Patients were censored at last follow-up if death status could not be determined or they remained alive at the time of analysis.

**Figure 3.**
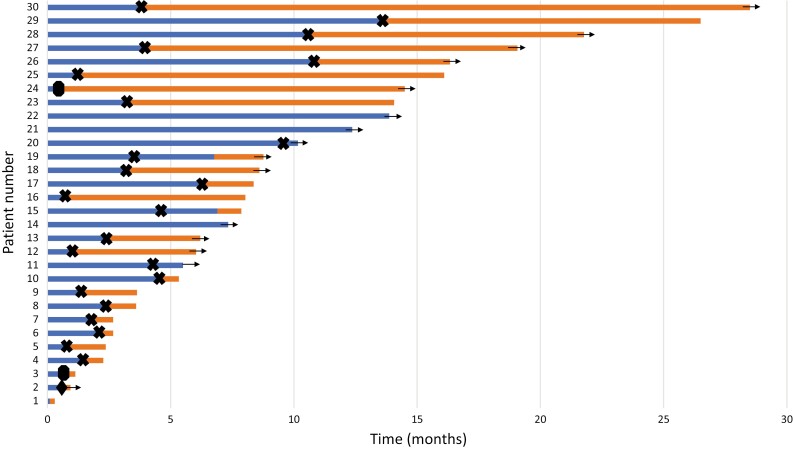
Swimmer plot showing time on treatment (dark blue) and overall survival (light orange) for patients receiving tivozanib. Arrows indicate patients who remained alive at the time of analysis. (X) indicates radiographic or clinical progression. Filled circle indicates treatment discontinuation due to toxicity. Filled diamond indicates treatment discontinuation for reasons other than progression or toxicity. Patients nos. 21 and 22 had confirmed partial response by RECIST v1.1. Patients nos. 11, 15, 19, and 20 continued treatment beyond progression. Patient no. 1 discontinued treatment due to toxicity.

Of the 30 patients in this cohort, 26 had follow-up imaging that allowed for evaluation of radiographic response by RECIST v1.1. Two patients achieved a PR (one patient had 34.5% tumor shrinkage and another patient had 65.7% tumor shrinkage from baseline) and no patients achieved a CR. The ORR for treatment with tivozanib was 2/26 (7.7%). The CBR was 7/30 (23.3%). With a median follow-up time of 13.9 months, the median PFS was 3.8 months (range, 0.7-13.9; Fig. 1). Median OS was 14.1 months (range, 0.3-28.5; Fig. 2); 15 patients had died by the time of analysis. Median TOT was 3.2 months (range, 0.1-13.9; Fig. 3). Four patients (13.3%) were continuing treatment with tivozanib at the time of analysis; 21 pts (70%) discontinued TIVO due to progressive disease, 3 pts (10%) for treatment-related adverse events, and 2 patients for other reasons (infection, worsening of prior immune checkpoint inhibitor-mediated neuropathy).

Patients with clinical benefit for at least 6 months are described in Table 2. 6/7 (85.7%) had IMDC intermediate-risk disease at the time of initiation of tivozanib. All patients had remained on a prior line of TKI therapy for at least 9 months. The time from start of first-line systemic therapy for metastatic disease to start of tivozanib ranged from 23 to 64 months.

**Table 2.** Description of patients with clinical benefit.

Patient number (Fig. 3)	ECOG performance status	IMDC risk score at TIVO treatment start	Prior therapies	Tivozanib starting daily dose (mg; 3 weeks on, 1 week off)	Best radiologic response	Time on tivozanib (months)	PFS (months)	OS from TIVO start (months)	Documented adverse events
22	2	2	1) Adjuvant sunitinib w/ disease progression during treatment 2) Nivolumab/ipilimumab (12 months) 3) Lung nodule RT 4) Cabozantinib (21 months)	1.34	PR	13.9	13.9 (ongoing)		None
28	1	2	1) Clinical trial (6 months) 2) Axitinib (9 months) 3) Clinical trial (9 months) 4) Lenvatinib/everolimus (5 months) 5) Clinical trial (8 months) 6) Clinical trial (3 months) [Patient received nivolumab and pembrolizumab as part of clinical trial protocols.]	1.34*	SD	10.6	10.6	21.8 (ongoing)	Grade 2 hypertension, fatigue, mucositis
26	1	2	1) Pazopanib (19 months) 2) Nivolumab (8 months) 3) Lung nodule RT 4) Observation (12 months) 5) Pembrolizumab (12 months) 6) Cabozantinib/nivolumab (13 months) 7) Lung nodule RT	0.89*	SD	10.8	10.8	16.3 (ongoing)	Grade 2 diarrhea
29	1	3	1) Bone lesion resection 2) Pazopanib (27 months) 3) Cabozantinib (9 months) 4) Axitinib/avelumab (2 months) 5) Lenvatinib/everolimus (8 months) 6) Nivolumab (3 months) 7) Adrenal lesions--RT and embolization	1.34*	SD	13.5	13.5	26.5	Grade 2 rash
21	0	2	1) Pazopanib (5 months) 2) Nivolumab/bevacizumab (27 months) 3) Axitinib/pembrolizumab (2 months) 4) Cabozantinib (15 months) 5) Clinical trial (8 months)	1.34	PR	12.4	12.4 (ongoing)		None
17	2	2	1) Bone lesion RT 2) Nivolumab/ipilimumab (7 months) 3) Cabozantinib (16 months) 4) Bone lesion RT	1.34*	SD	6.3	6.3	8.4	Grade 2 fatigue, stomatitis
20	0	1	1) Pembrolizumab (2 months) 2) Axitinib (24 months)	1.34	SD	10.2	9.4	10.2 (ongoing)	Grade 1 fatigue

Characteristics and treatment courses for patients who had clinical benefit for at least 6 months with tivozanib. Asterisks indicate patients who required dose reduction. Patients still alive at the time of analysis are indicated as “ongoing” for OS. Patient no. 17 continued tivozanib despite clinical progression.

Fifteen (50%) patients received subsequent therapies after discontinuation of tivozanib. Subsequent therapies were belzutifan (5 patients), lenvatinib ± everolimus (4 patients), clinical trials (3 patients), cabozantinib ± nivolumab (2 patients), and nivolumab (one patient).

### Toxicity

Fifteen (50%) patients had at least one treatment-related adverse event documented (Table 3). Six (20%) patients experienced a grade 3 or higher treatment-related adverse event. These events were GI perforation (grade 5), congestive heart failure (3 events, one grade 3 and 2 grade 4), hypertension (grade 3), and mucositis (grade 3). Four (13.3%) patients had documentation of any-grade hypertension, 4 patients (13.3%) had any-grade fatigue, 3 (10%) had diarrhea/abdominal pain, and 3 (10%) had mucositis/stomatitis. Seven patients (23.3%; 5 patients who started at 1.34 mg/day and 2 patients who started at 0.89 mg/day) required dose-reduction due to treatment related adverse events. Both patients who had started at the 0.89 mg/day dose level were decreased from 3 weeks on, 1 week off to 2 weeks on, 2 weeks off.

**Table 3.** Treatment-related adverse events.

Adverse event, *n* (%)	All grades	Grade 3 or higher
Any events	15 (50)	6 (20)
Hypertension	4 (13.3)	1 (3.3)
Fatigue	4 (13.3)	0 (0)
Mucositis/stomatitis	3 (10)	1 (3.3)
Congestive heart failure	3 (10)	3 (10)
Diarrhea	2 (6.7)	0 (0)
Bowel perforation	1 (3.3)	1 (3.3)
Hypothyroidism	1 (3.3)	0 (0)
Rash	1 (3.3)	0 (0)
Hand-foot syndrome	1 (3.3)	0 (0)
Abdominal pain	1 (3.3)	0 (0)
Anorexia	1 (3.3)	0 (0)

Treatment-related adverse events documented among patients treated with tivozanib. The total number of events equals more than 15 because some patients had multiple events.

## Discussion

In this group of heavily pretreated patients with clear cell RCC, the clinical benefit of tivozanib was limited, with ORR (7.7%) and median PFS (3.8 months), both substantially lower than in the TIVO-3 trial (ORR: 18%; median PFS: 5.6 months). Almost all patients in this analysis had been treated with a broader-spectrum TKI such as cabozantinib (which targets VEGFR1/2/3, MET, Kit, Flt-3, Tie-2, TrkB, and Axl) and/or lenvatinib (which targets VEGFR2, FGFR1/2/3/4, PDGFRα, Kit, and Ret), suggesting that patients with disease progression on those agents are unlikely to benefit from tivozanib, which has a narrower spectrum of activity and targets predominantly the VEGF receptors.^1^

It should be noted that the ORR, CBR (23.3%), and median PFS for tivozanib in this study are significantly lower than reported with other TKIs in later-line treatment of RCC. One retrospective study examined lenvatinib ± everolimus in patients with a median of 4 prior lines of therapy. In the cohort of 45 patients with clear cell RCC, the ORR was 24.4% and median PFS 7.1 months, with over 85% of patients achieving SD by RECIST v1.1 criteria.^10^ Similarly, in a multicenter retrospective analysis of patients treated with any TKI after prior IO, the aggregate ORR for TKI therapy in the 4th or later line setting was 31.1% with median time to treatment discontinuation of 6.1 months.^11^ Less than 30% of patients in that analysis had IMDC poor-risk disease, and blinded radiology review was not incorporated. Both of those factors may have led to a higher reported clinical benefit than what we observed for tivozanib. All these data contrast significantly with the reported benefit of TKI-based therapy in the 2nd line setting immediately following immune checkpoint inhibitor treatment, where response rates above 40% have been reported for multiple agents.^12-14^ Most notably, the recently published phase III CONTACT-03 trial compared cabozantinib monotherapy versus the combination of cabozantinib and atezolizumab in patients with disease progression on ICT. Most patients had received one prior VEGFR-targeting TKI. The median PFS and ORR in the cabozantinib monotherapy arm were 10.8 months and 41%, respectively, which are substantially higher than observed for tivozanib in our study.^15^ The efficacy of tivozanib (either alone or in combination with nivolumab) in the second- or third-line setting after prior ICT is currently being evaluated in phase III, randomized TiNivo-2 trial.^16^ In the single-arm, phase Ib/II TiNivo trial, which included 48% treatment-naive patients, the ORR for tivozanib plus nivolumab was 56%.^17^

There are several noteworthy observations upon closer examination of the 7 patients who demonstrated clinical benefit for at least 6 months with tivozanib therapy. First, these patients had previous history of prolonged duration of therapy with at least one prior TKI, indicating disease biology that was responsive to VEGF inhibition. Six of the patients remained on at least one prior TKI for over 12 months, and 2 patients had prolonged durations (19 and 27 months) of treatment with pazopanib. The patients with clinical benefit had relatively indolent metastatic disease, having started systemic therapy for metastatic RCC between 23 and 64 months prior to the start of tivozanib therapy. Only one of the patients with clinical benefit from tivozanib had IMDC poor-risk disease at treatment start, further supporting the concept that the benefit of tivozanib is limited to patients with more indolent disease.

The toxicity profile of tivozanib was in line with other agents that target the VEGF pathway. Overall, we noted a preponderance of cardiovascular events, particularly congestive heart failure (3 grade ≥ 3 events). Two of those cases occurred in patients with pre-existing, but well-managed congestive heart failure, signaling a need for significant caution with administration of tivozanib in this population. Four of the patients with clinical benefit from tivozanib required a dose-reduction due to adverse events, including one patient to off-label dosing of 0.89 mg/day, 2 weeks on, 2 weeks off. This indicates that tivozanib is still active and can provide clinical benefit below the full dose. The frequency of grade 3 or higher adverse events was similar to the CBR for the cohort and higher than the ORR. This further reinforces the limited balance of risk versus benefit and overall clinical utility of tivozanib in this setting.

The recently announced results of the LITESPARK-005 trial and FDA approval of belzutifan,^18^ which has a different mechanism of action (HIF-2 alpha inhibition) compared with VEGFR-targeted TKIs, add new uncertainty regarding the role of tivozanib in management of advanced RCC. For patients who have received dual-ICT or ICT-TKI combination therapy in the front-line, followed by a broad-spectrum TKI such as cabozantinib or lenvatinib in the second-line, belzutifan offers an advantage over tivozanib in providing a reprieve from the chronic and cumulative toxicities of TKI therapy.^19^ The efficacy of tivozanib in patients previously treated with belzutifan, or vice versa, is a question that merits further exploration in future studies.

## Conclusion

The efficacy of tivozanib in advanced clear cell RCC is limited among patients who are heavily pretreated. Hypertension and congestive heart failure were the most prevalent adverse events in our study cohort, and based on our experience, tivozanib should be used with caution in patients with pre-existing cardiovascular disease, even if it is well-managed. Tivozanib is an appropriate agent for well-selected patients with relatively indolent advanced RCC and prior prolonged therapy with other TKIs. Patients with rapidly progressive disease or aggressive disease biology are less likely to benefit from this therapy.

## Data Availability

The data underlying this article will be shared on reasonable request to the corresponding author.

## Prior Presentation

Portions of the data in this article were previously presented at the International Kidney Cancer Symposium: North America in Nashville, TN, November 9-11, 2023, and at the ASCO Genitourinary Cancers Symposium in San Francisco, CA, January 25-27, 2024.
